# 6-Year Periodicity and Variable Synchronicity in a Mass-Flowering Plant

**DOI:** 10.1371/journal.pone.0028140

**Published:** 2011-12-07

**Authors:** Satoshi Kakishima, Jin Yoshimura, Hiroko Murata, Jin Murata

**Affiliations:** 1 Botanical Gardens, Graduate School of Science, the University of Tokyo, Bunkyo, Tokyo, Japan; 2 Department of Systems Engineering, Shizuoka University, Hamamatsu, Shizuoka, Japan; 3 Department of Environmental and Forest Biology, State University of New York College of Environmental Science and Forestry, Syracuse, New York, United States of America; 4 Marine Biosystems Research Center, Chiba University, Kamogawa, Chiba, Japan; 5 Faculty of Pharmaceutical Sciences, Setsunan University, Hirakata, Osaka, Japan; University of Zurich, Switzerland

## Abstract

Periodical organisms, such as bamboos and periodical cicadas, are very famous for their synchronous reproduction. In bamboos and other periodical plants, the synchronicity of mass-flowering and withering has been often reported indicating these species are monocarpic (semelparous) species. Therefore, synchronicity and periodicity are often suspected to be fairly tightly coupled traits in these periodical plants. We investigate the periodicity and synchronicity of *Strobilanthes flexicaulis*, and a closely related species *S. tashiroi* on Okinawa Island, Japan. The genus *Strobilanthes* is known for several periodical species. Based on 32-year observational data, we confirmed that *S. flexicaulis* is 6-year periodical mass-flowering monocarpic plant. All the flowering plants had died after flowering. In contrast, we found that *S. tashiroi* is a polycarpic perennial with no mass-flowering from three-year individual tracking. We also surveyed six local populations of *S. flexicaulis* and found variation in the synchronicity from four highly synchronized populations (>98% of plants flowering in the mass year) to two less synchronized one with 11–47% of plants flowering before and after the mass year. This result might imply that synchrony may be selected for when periodicity is established in monocarpic species. We found the selective advantages for mass-flowering in pollinator activities and predator satiation. The current results suggest that the periodical *S. flexicaulis* might have evolved periodicity from a non-periodical close relative. The current report should become a key finding for understanding the evolution of periodical plants.

## Introduction

Periodical organisms are very famous for their mass and synchronous reproduction, e.g., bamboos [1]–[3], periodical cicadas [4]–[6]. The characteristic features of these organisms are the periodicity and synchronicity of reproductive events, e.g., periodical mass-flowering in bamboos [1]–[3]. Even though periodicity and synchronicity should be considered essentially two separate phenotypic traits, periodical organisms are usually associated with both periodicity and synchronicity [5], [6]. Therefore, it is particularly important to investigate the evolutionary backgrounds of periodicity and synchronicity in these periodical organisms. Specifically, in periodical mass-flowering monocarpic (semelparous) plants, synchronous flowering is the key factor for pollination and seed predation avoidance [1], [7], [8].

However, it is highly difficult, if not impossible, to investigate the relationship between periodicity and synchronicity in many periodical plants, because most these plants have extremely long life cycles (e.g., 15–120 years in bamboos). Based on the extensive studies, the confirmation of periodicity in periodical cicadas has been widely accepted [4]–[6]. However, the confirmation of periodicity in bamboos is still highly difficult [1]. It is also further difficult to evaluate whether these organisms are perfectly synchronized or not even from extensive field surveys. In bamboo species, particularly, the synchronicity of individual plants is almost impossible because we cannot identify the vegetative grown genets from other individuals [2].

Instead of studying these long-cycle periodical organisms, we here investigated a periodical plant with relatively shorter life cycles (much less than 10 years). In the genus *Strobilanthes* (Acanthaceae), many species are reported to have periodicity with shorter life cycles, i.e., mass-flowering with various synchronous cycles (3–16 years), while other species flower every year [1], [9], [10]. We investigated the periodicity and synchronicity in supposedly periodical *S. flexicaulis* and its closely related *S. tashiroi*, both occurring on Okinawa Island, in the Ryukyu Islands, Japan. We also investigate the pollinator activities and predator satiation in *S. flexicaulis* to examine the selective advantages of mass-flowering.

## Results

**Figure 1:**
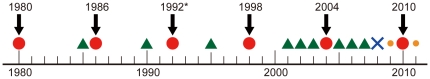
Mass-flowering years of *Strobilanthes flexicaulis*. Flowering records of *Strobilanthes flexicaulis* at Mt. Yae and Mt. Katsuu are shown as mass flowering (large red circles), few flowers (small orange circles), no flowers (blue crosses), and non-mass-flowering (green triangles), when we have no records of flower counts. *This mass-flowering was observed by K. Nakajima (personal communication).

Field observations were carried out at Mt. Katsuu and Mt. Yae in the Motobu Peninsula, Okinawa Island from 1980. Mass-flowering of *Strobilanthes flexicaulis* was recorded at a six-year cycle in 1980, 1986, 1992, 1998, 2004, and 2010, while the rest 12 years were either few flowers or no flowers (Fig. 1). The seedling experiments starting in 1998, 2004 and 2005 showed that 17 out of 20 seedlings flowered (and withered) at six years, and the rest three plants at seven years (Table 1). Therefore, the interval of periodical mass-flowering had been estimated six years.

**Table 1:** Seedling experiments.

sowing year	individuals	6th year	7th year
1998	9	9	0
2004	8	5	3
2005	3	3	0

The seedling experiments starting in 1998, 2004 and 2005 were performed in the greenhouse in Medicinal Botanical Garden, Faculty of Pharmaceutical Sciences, Setsunan University. The numbers of flowering individuals in each year were shown.

Detailed examinations of the life history for *S. flexicaulis* were performed from 2008 to 2011 (Table 2). We surveyed all flowering individuals in populations at Mt. Iyu, Mt. Oppa, and Awa. In all six populations, the number of flowering individuals was highest in 2010, indicating mass-flowering. Only few flowering plants are seen in 2009 or/and 2011 in four out of six populations (Mt. Iyu, Mt. Katsuu, Mt. Yae and Mt. Oppa). The populations at Mt. Yae and Mt. Katsuu show high synchronicity, more than 99% individuals flowered in 2010 with only a few one-year displacements (*χ^2^* = 1.30, df = 1, *P* = 0.25; Text S1 and Table S1). Note that we exclude Mt. Iyu and Mt. Oppa from the current comparisons, since the numbers of plants at Mt. Iyu and Mt. Oppa are too small for statistical inference. In the other two populations (Mt. Nago and Awa), relatively large numbers of plants were flowered in before/after 2010. Thus the synchronicity is much lower at Mt. Nago and Awa compared with the other four localities. The chi-square test shows that the synchronicity of Mt. Nago is significantly lower than that of Mt. Katsuu and Mt. Yae (*χ^2^* = 234.48, df = 2, *P*<0.01; Text S1, Table S2). Furthermore, in Awa population, the number of flowering plants in 2011 (31 individuals) is about the same with that in 2010 (34 individuals) and 25 individuals are still not flowering at 2011 that are expected to flower next year if survived. The chi-square test shows that the synchronicity of Awa is significantly lower than that of Mt. Katsuu and Mt. Yae (*χ^2^* = 827.58, df = 2, *P*<0.01; Text S1, Table S3). Thus the flowering years are spread almost three consecutive years at Mt. Nago and Awa, showing low levels of synchronicity.

**Table 2:** Numbers of flowering individuals and survived individuals.

population		2008	2009	2010	2011
Mt. Iyu	flowering	–	0	15	0
	survived/labeled	–	–	0/15	–
Mt. Katsuu	flowering*	0	11	1882	0
	survived/labeled	–	0/11	0/21	–
Mt. Yae	flowering*	0	9	1043	1
	survived/labeled	–	4/9	0/102	–
Mt. Oppa	flowering	–	1	>50	0
	survived/labeled	–	–	0/18	–
Mt. Nago	flowering*	13	6	484	45
	survived/labeled	0/13	0/6	0/30	–
Awa	flowering*	–	0	34	31
	survived/labeled	–	–	0/16	–

The numbers of flowering individuals for *Strobilanthes flexicaulis* were enumerated in six populations from 2008 to 2011. We surveyed all flowering individuals at Mt. Iyu, Mt. Oppa, and Awa. The number of survived individuals/labeled individuals is shown in the second row of each locality, where some flowering plants were labeled and their survivals were examined in the following year.

*The chi-square test shows that the synchronicity of Mt. Nago and Awa was significantly lower than Mt. Katsuu and Mt. Yae (Text S1).

In the total of six populations, we keep track of 241 plants by individual labeling (Table S4). Out of the 241 individuals, 237 died after flowering (Table 2). The remaining four individuals, in Mt. Yae population, had flowered partially on some branches. These branches had withered after flowering. These all four plants then had flowered in the following year and died after. Thus the death after flowering is a stable phenotypic character in *S. flexicaulis* even at the level of branches.

**Figure 2:**
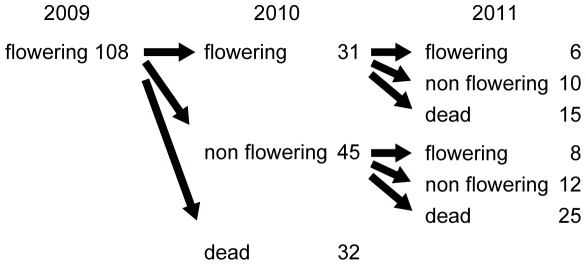
Life history of *Strobilanthes tashiroi*. One hundred eight flowering individuals of *Strobilanthes tashiroi* were labeled in 2009. These individuals were pursued until 2011 and examined conditions (flowering, non flowering or dead) in each year. Three year consecutive flowering in *S. tashiori* clearly shows it is a polycarpic perennial plant.

Comparing with *S. flexicaulis*, mass-flowering has never been observed in *S. tashiroi*. In the total of three populations, we labeled 108 individuals of *S. tashiroi* (Table S4). In 2010, 76 labeled individuals were alive and the other 32 individuals were dead (Fig. 2). Out of the 76 individuals, 31 flowered again. In 2011, 36 individuals survived and the other 40 individuals died. Six individuals bloomed for three consecutive years.

We also compare pollinator activities with *S. flexicaulis* and *S. tashiroi* between the mass-flowering year and the previous/following years (Table 3). Even though these species flowered in the winter season, in the mass-flowering year, three insect pollinators are seen (1.71 individuals/hour): humming-bird hawk moths (*Macroglossum corythus platyxanthum*, 0.46 individuals/hour); honeybees (*Apis mellifela*, 1.18 individuals/hour); and butterflies (*Byasa alcinous loochooana*, 0.07 individuals/hour). In contrast, in the year before/after mass-flowering year, the same humming-bird hawk moth was observed to make a single visit during the total observation of 13.25 hours (0.09 individuals/hour). Thus the level of insect pollinations is extremely low.

**Table 3:** Pollinator observations.

year	time (hour)	*Macroglossum corythus platyxanthum*	*Apis mellifera*	*Byasa alcinous loochooana*
2009	10.75	0.09	0.00	0.00
2010	15.25	0.46	1.18	0.07
2011	2.5	0.00	0.00	0.00

Pollinator activities (observed individuals/hour) in Mt. Yae population before/during/after mass-flowering year are shown.

**Figure 3:**
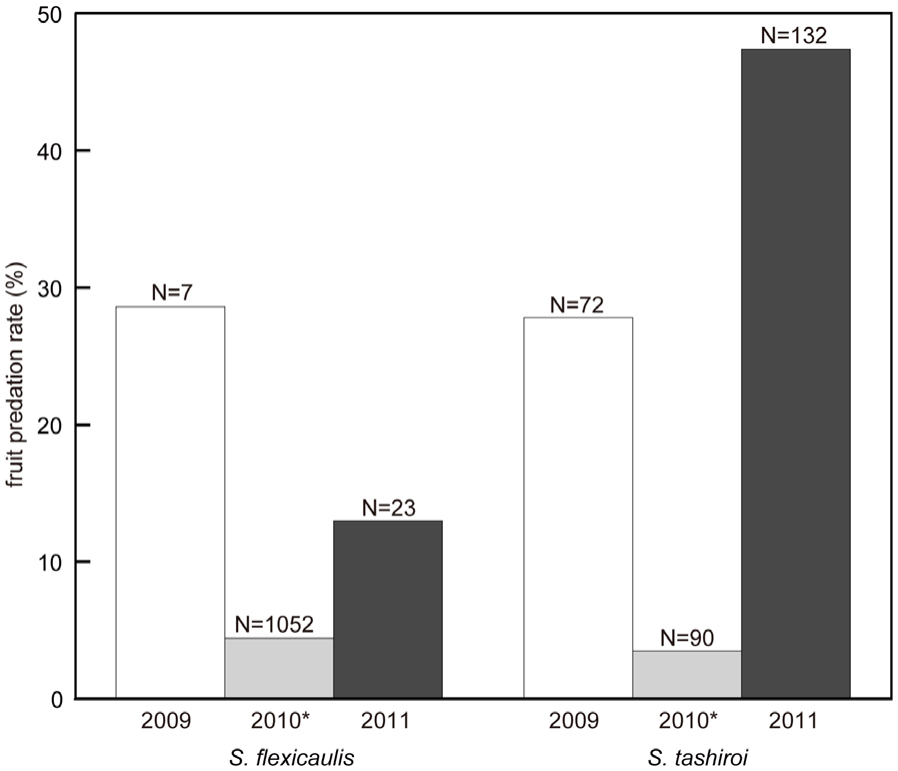
Surveys of fruit predation rates. All the fruits on a plant before the capsules were exploded, were examined in Mt. Yae population at the end of March or the beginning of April from 2009 to 2011. The number of flowers examined was shown on each bar. * Fruit predation rates in the mass-flowering year (2010) were significantly low with the chi-square test (Text S3).

In order to evaluate the effects of predator satiation on the selective advantages of mass-flowering, we compared the percentage of fruits predated in *S. flexicaulis* and *S. tashiroi* between the mass-flowering year and the previous/following years (Fig. 3). Fruit predation by the larvae of Pterophoridae sp. was observed in Mt. Yae population. Fruit predation rates in the mass-flowering year (2010) were significantly low in both *S. flexicaulis* (*χ^2^* = 13.5, df = 1, *P*<0.01; Text S3, Table S5) and *S. tashiroi* (*χ^2^* = 39.4, df = 1, *P*<0.01; Text S3, Table S6) with the chi-square test.

## Discussion

From 32-year data, it is confirmed that *Strobilanthes flexicaulis* is a periodical plant with a six-year mass-flowering cycle (Fig. 1). Detailed observations of six populations during 2008 and 2011 revealed that all plants (branches) had died after flowering (Table 2). These results suggested that *S. flexicaulis* was a periodical plant that maintained a periodical mass-flowering on Okinawa Island. However, the synchronicity of flowering is variable among local populations (Table 2). Mt. Iyu, Mt. Yae, Mt. Katsuu and Mt. Oppa show high levels of synchrony, while there is much lower synchrony at Nago and Awa. These results indicate that the synchronicity in *S. flexicaulis* is relatively variable depending on the locality. Thus *S. flexicaulis* is confirmed a monocarpic periodical plant as in bamboos, but with some variation in the levels of synchronization.

Unlike *S. flexicaulis*, *S. tashiroi* was confirmed to be a polycarpic perennial plant; each plant produces flowers at least for several years without withering (Fig. 2). No mass-flowering had been observed in *S. tashiroi*. Both *S. flexicaulis* and *S. tashiroi* belong to Parachampionella group, a small closely related taxonomic group consisting of four species [11], [12]. These two species are genetically closely related [11]. This means that two distinctively different life histories, a polycarpic perennial plant and a monocarpic periodical plant, have been occurred in a small closely related taxonomic group. From the uniqueness and rarity of monocarpic periodical life history, we suspect that perennial life history is the ancestral trait for monocarpic periodical life history. Because of its shorter life cycle, and the variation of life history in its taxonomic group, *S. flexicaulis* could be a good model organism for the study of the evolution of monocarpic periodical life history, and periodical plants in general [1].

For the evolution of periodicity and synchrony we need to evaluate two traits: (1) an internal clock, set for six years in this case, and (2) a selection to keep populations (cohorts) synchronous [1]. The latter synchrony comes from lower fitness of out-of-step plants (from higher selective mortality of seeds produced in off years, or from failure to pollinate if flowering in off years). A rarer, second out-of-step cohort growing at the same site, which would be expected to arise easily enough, would be easily eliminated due to lower fitness in the same manner, if selection favors synchrony [1].

It is important to consider the evolutionary relationships between periodicity and synchrony [1]. Even though periodicity and synchrony are separate traits, masting or mass-flowering should be achieved by simultaneous evolution of synchrony and periodicity to some extent [7], [8]. Koenig et al. [13] showed some selective advantages of simultaneous periodicity and synchrony in iteroparous plant species. Such selective advantages should be much higher in semelparous species with strict masting [7], since all the adult plants die after flowering (reproduction), e.g., Bamboos [1], *Cerberiopsis* [14], [15], *Isoglossa* [16], *Stenostephanus* [9], *Strobilanthes* [10], *Tachigali* [17], [18]. As Janzen [1] pointed out, outliers in strict masting are usually common, i.e., out of periods and asynchronous flowering. The current recorded data of *S. flexicaulis* shows many off-year flowerings. Our seedling experiments also show three out of 20 plants with one-year delay. Thus, in the current case the six-year periodicity is relatively well established, while synchrony still involves variation depending on the locality. We therefore suspect that the evolution of periodicity might have preceded the selection for synchrony in *S. flexicaulis*. There are several factors affecting the reproductive success of mass-flowering, e.g., pollination, seed predation [1], [7], [8].

Mass-flowering may increase the efficiency of pollination [1], [7]. It is often suggested that mass-flowering is highly effective in wind pollination [8], [19]. In contrast, mass-flowering is suggested to have less or no effect under insect pollination [8], [20]. Interestingly the current case might be an exception in insect pollination [7], since this plant flowers in winter. Winter flowers have extremely few insect pollinators, often resulting in no pollinators in isolated flowers. We observed the number of pollinators in mass-flowering monocarpic *S. flexicaulis* and related polycarpic *S. tashiroi* before/during/after mass-flowering year at the same locality (Table 3). We find almost no pollinators before and after mass-flowering, but many pollinators during mass-flowering. Pollinator activity may be highly crucial in winter flowering when the number of pollinators is extremely low. Mass-flowering may be able to attract these few pollinators; otherwise impossible.

Seed predation might be lowered significantly with masting [1], [7], [17], [21], [22]. We compare the rates of fruit predation in mass-flowering monocarpic *S. flexicaulis* and related polycarpic *S. tashiroi* before/during/after mass-flowering year at the same locality (Fig. 3). In both *S. flexicaulis* and *S. tashiroi*, predation rates are low when mass-flowering, but much higher a year before and after. This suggests that mass-flowering at least reduces the level of predation considerably. Thus the selection favoring synchronicity may be promoted by pollinator activities [7], [23] and predator avoidance [1], [7].

The lack of synchrony in some populations may be basically errors by individual plants, e.g., deleterious mutations and recombination. These out-of-synchronous plants should be weeded out by natural selection later. The current variation in the level of synchrony may be a result of such stochastic factors in small populations and intermittent errors in plant cycle length, that have been appearing temporally before the selection weeds out.

## Materials and Methods

### Ethics Statement

We were permitted to collect and observe in the Okinawakaigan Quasi-National Park from the Governor of Okinawa, and the Natural Conservation Zone of Mt. Katsuu, Mt. Awa and Mt. Yae from the Okinawa Prefectural Board of Education.

### Species and morphology

*Strobilanthes flexicaulis* is a subshrub distributed in the Ryukyu Islands, Japan, and Taiwan [12]. *Strobilanthes tashiroi* is a perennial herb endemic to the Ryukyu Islands [12]. These species are closely related and morphologically very similar [12], [24]. Around the Motobu peninsula on Okinawa Island in the Ryukyu Islands, *S. flexicaulis* and *S. tashiroi* grow sympatrically. We identified these species based on the bract shape and the difference in length between longer (anterior) and shorter (posterior) pairs of stamens, which are didynamous (Text S2, Figure S1, Table S4).

### Study sites and observation of life histories

We recorded mass-flowering years of *S. flexicaulis* and *S. tashiroi* around the Motobu Peninsula on Okinawa Island from 1980 to 2011. Quantitative research of mass-flowering species was performed in six populations (Table S4). All flowering individuals of *S. flexicaulis* were enumerated in the definite zones along paths at Mt. Katsuu, Mt. Yae, and Mt. Nago from 2008 to 2011, and at Awa, Mt. Oppa, and Mt. Iyu from 2009 to 2011. The difference in the synchronicity among populations was checked by the chi-square test (Text S1). Some representative flowering individuals of *S. flexicaulis* were labeled in winter from 2008 to 2010 (Table S4). After flowering, life or death of labeled individuals was checked in the following summer. We labeled some flowering individuals of *S. tashiroi* at Mt. Nishime, Mt. Yae and Mt. Nago in 2008 and checked whether these labeled individuals survived and flowered, survived but did not flower, or died in the two successive years, 2009–2010 (Table S4). The seedling experiments starting in 1998, 2004 and 2005 were performed in the greenhouse in Medicinal Botanical Garden, Faculty of Pharmaceutical Sciences, Setsunan University (Hirakata, Japan). A total of 20 individuals were sown and cultivated until flowering and dying.

### Pollinator activities and fruit predation

Pollinator observations and surveys of fruit predation rates are performed in high synchronous Mt. Yae population from 2009 to 2011. Because pollinators were not seen in nighttime in preliminary observations, we observed pollinators in daytime in mostly *S. flexicaulis* in the mass-flowering year and mostly *S. tashiroi* in the previous and following years, as these are the available flowers in these years, respectively. To evaluate the fruit predation rates, we examined all the fruits on a plant before the capsules were exploded, at the end of March or the beginning of April from 2009 to 2011. The difference in the fruit predation rates in each species between the mass-flowering year and before/after was checked by the chi-square test (Text S3).

## Supporting Information

Text S1 The chi-square test was performed to examine the difference in synchronicity among populations.
(DOC)

Text S2 *Strobilanthes flexicaulis* and *S. tashiroi* were identified by the combination of the bract shape and the difference in length between longer and shorter pairs of stamens.
(DOC)

Text S3 The chi-square test was performed to examine the difference in fruit predation rates between the mass-flowering year and off years.
(DOC)

Figure S1 The bract shape (length/width) and the difference in length between longer and shorter pairs of stamens are shown. Blue circles are *Strobilanthes flexicaulis* individuals and red squares are *S. tashiroi*.
(TIF)

Table S1 The contingency table for the chi-square test to examine the difference in synchronicity between Mt. Yae and Mt. Katsuu.
(XLS)

Table S2 The contingency table for the chi-square test to examine the difference in synchronicity between Mt. Yae, Mt. Katsuu and Mt. Nago.
(XLS)

Table S3 The contingency table for the chi-square test to examine the difference in synchronicity between Mt. Yae, Mt. Katsuu and Awa.
(XLS)

Table S4 Localities and geographical coordinates of observed or/and sampling populations, where the extent of observed areas in each population and the sampling numbers of *Strobilanthes flexicaulis* and *S. tashiroi* in pure populations are shown.
(XLS)

Table S5 The contingency table for the chi-square test to examine the difference in fruit predation rate between the mass-flowering year and off years in *Strobilanthes flexicaulis*.
(XLS)

Table S6 The contingency table for the chi-square test to examine the difference in fruit predation rate between the mass-flowering year and off years in *Strobilanthes tashiroi*.
(XLS)
