# Supplementary material for: 6-Year Periodicity and Variable Synchronicity in a Mass-Flowering Plant
Source: PLoS One. 2011 Dec 7;6(12):e28140. doi: 10.1371/journal.pone.0028140 (PMC3233548; doi:10.1371/journal.pone.0028140)
Supplement: Text S1 — The chi-square test was performed to examine the difference in synchronicity among populations. (DOC) [file pone.0028140.s001.doc]

**Text S1.**

The chi-square test was performed to examine the difference in synchronicity among populations. First, we performed a 2 x 2 contingency chi-square test to examine the difference in synchronicity between Mt. Yae and Mt. Katsuu that showed high synchronicity (Table S2). One variable was populations and the other variable was years that are the mass-flowering year (2011) and off years (2008+2009+2011). The difference in synchronicity between Mt. Yae and Mt. Katsuu is not detected significantly (*χ^2^*=1.30, df=1, *P*=0.25). Second, we performed a 3 x 2 contingency chi-square test to examine the difference in synchronicity between Mt. Yae, Mt. Katsuu and Mt. Nago (Table S3). Two categories of years were the mass-flowering year (2011) and off years (2008+2009+2011). The chi-square test shows that the synchronicity of Mt. Nago is significantly lower than that of Mt. Katsuu and Mt. Yae (*χ^2^*=234.48, df=2, *P*<0.01). Third, we performed a 3 x 2 contingency chi-square test to examine the difference in synchronicity between Mt. Yae, Mt. Katsuu and Awa (Table S4). Two categories of years were the mass-flowering year (2011) and off years (2009+2011). The chi-square test shows that the synchronicity of Awa is significantly lower than that of Mt. Katsuu and Mt. Yae (*χ^2^*=827.58, df=2, *P*<0.01).
