# Supplementary material for: 6-Year Periodicity and Variable Synchronicity in a Mass-Flowering Plant
Source: PLoS One. 2011 Dec 7;6(12):e28140. doi: 10.1371/journal.pone.0028140 (PMC3233548; doi:10.1371/journal.pone.0028140)
Supplement: Text S2 — Strobilanthes flexicaulis and S. tashiroi were identified by the combination of the bract shape and the difference in length between longer and shorter pairs of stamens. (DOC) [file pone.0028140.s002.doc]

**Text S2.**

Although *Strobilanthes flexicaulis* and *S. tashiroi* are easily distinguishable by pollen morphology (Wood and Scotland, 2003), the examination of pollen morphology was actually difficult in natural populations. Wood and Scotland (2003) indicated that bract morphology was also different: bracts of *S. flexicaulis* were broadly ovate and those of *S. tashiroi* were linear. In addition, from our preliminary observations, it was predicted that the difference in length between longer and shorter pairs of stamens was bigger in *S. flexicaulis*. We measured the length and width of a bract, and the length of longer and shorter pairs of stamens. Forty-three individuals were collected from Mt. Katsuu and Awa, which were pure populations of *S. flexicaulis*, and 68 individuals were collected from six pure populations of *S. tashiroi* (Table S1). To compare the bract shape of *S. flexicaulis* with that of *S. tashiroi,* we calculated the ratio of a bract (the length divided by the width). The maximum value of *S. flexicaulis* was 1.65 and the minimum of *S. tashiroi* was 1.64 (Fig. S1). The difference in length between longer and shorter pairs of stamens in *S. flexicaulis* was bigger than that in *S. tashiroi*. The minimum length of *S. flexicaulis* was 4.80 and the maximum of *S. tashiroi* was 3.55 (Fig. S1). These species are identified by the combination of the bract shape and the difference in length between longer and shorter pairs of stamens without any ambiguity.
