# Supplementary material for: 6-Year Periodicity and Variable Synchronicity in a Mass-Flowering Plant
Source: PLoS One. 2011 Dec 7;6(12):e28140. doi: 10.1371/journal.pone.0028140 (PMC3233548; doi:10.1371/journal.pone.0028140)
Supplement: Text S3 — The chi-square test was performed to examine the difference in fruit predation rates between the mass-flowering year and off years. (DOC) [file pone.0028140.s003.doc]

**Text S3.**

We performed a 2 x 2 contingency chi-square test to examine the difference in fruit predation rates between the mass-flowering year and off years. One variable was fruit predation and the other variable was years that are the mass-flowering year (2011) and off years (2009+2011). A fruit predation rate in the mass-flowering year was significantly low in *Strobilanthes flexicaulis* (*χ^2^*=13.5, df=1, *P*<0.01; Table S5). Similarly, the fruit predation rate of *S. tashiroi* in the mass-flowering year was significantly low (*χ^2^*=39.4, df=1, *P*<0.01; Table S6).
