# Supplementary figures and images for: 6-Year Periodicity and Variable Synchronicity in a Mass-Flowering Plant
Source: PLoS One. 2011 Dec 7;6(12):e28140. doi: 10.1371/journal.pone.0028140 (PMC3233548; doi:10.1371/journal.pone.0028140)

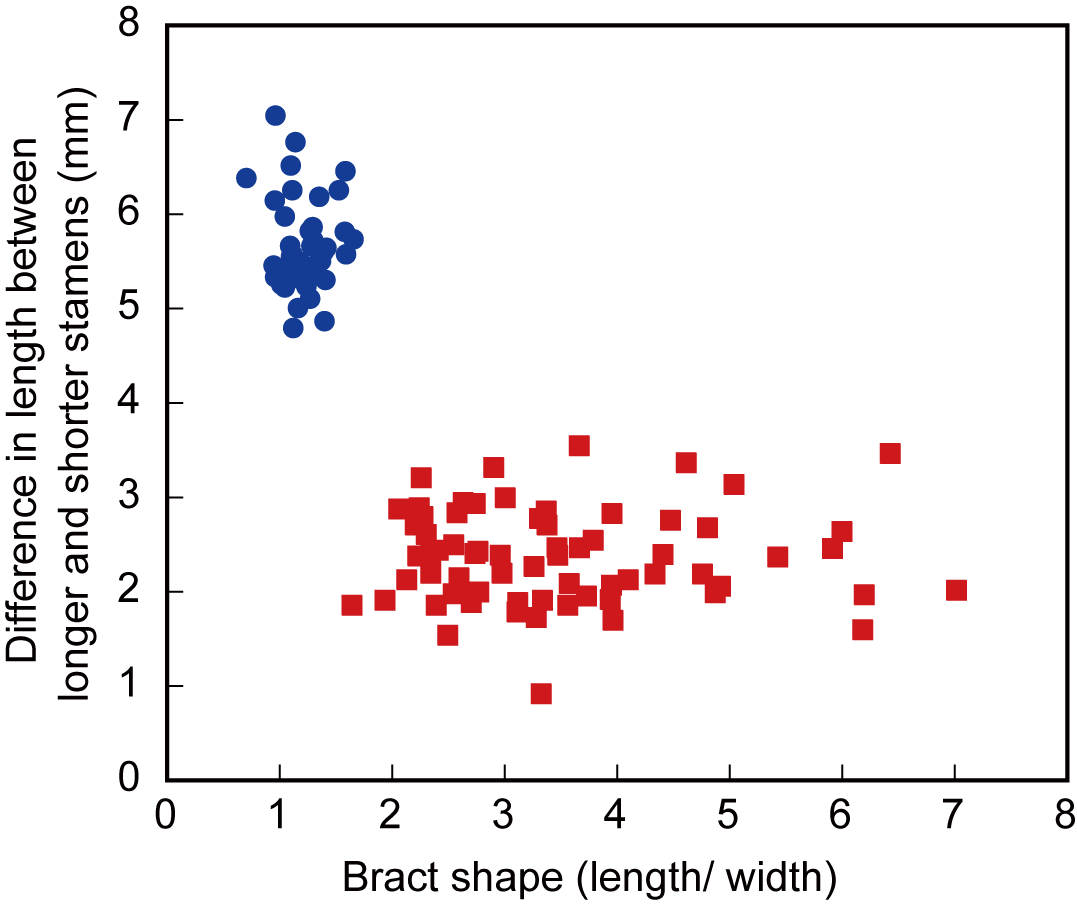

Supplement: Figure S1 — The bract shape (length/width) and the difference in length between longer and shorter pairs of stamens are shown. Blue circles are Strobilanthes flexicaulis individuals and red squares are S. tashiroi. (TIF) [file pone.0028140.s004.tif]
